# Are our babies off to a healthy start? The state of implementation of the *Global strategy for infant and young child feeding* in Europe

**DOI:** 10.1186/s13006-020-00282-z

**Published:** 2020-06-04

**Authors:** Irena Zakarija-Grković, Adriano Cattaneo, Maria Enrica Bettinelli, Claudia Pilato, Charlene Vassallo, Mariella Borg Buontempo, Helen Gray, Clare Meynell, Patricia Wise, Susanna Harutyunyan, Stefanie Rosin, Andrea Hemmelmayr, Daiva Šniukaitė-Adner, Maryse Arendt, Arun Gupta

**Affiliations:** 1 University of Split School of Medicine, Šoltanska 2, 21000 Split, Croatia; 2 IBFAN Italia, Androna San Fortunato 8, 34136 Trieste, Italy; 3 IBFAN Italia and University of Milan, School of Medicine, Via Festa del Perdono 7, 20122 Milan, Italy; 4 IBFAN Italia, Via Noce 20, 90138 Palermo, Italy; 5 Health Promotion and Disease Prevention Directorate, St Luke’s Square, Guardamangia, PTA 1010, Pietà, Malta; 6 Lactation Consultants of Great Britain, 6 Livingstone Terrace, Bath, BA2 3LE UK; 7 NCT, 30 Euston Square, London, NW1 2FB UK; 8 Yerevan State Medical University after Mkhitar Heratsi, 2 Koruni str, 0025 Yerevan, Armenia; 9 Charité – Universitätsmedizin Berlin, corporate member of Freie Universität Berlin, Humboldt-Universität zu Berlin, and Berlin Institute of Health, Klinik für Neonatologie, Campus Virchow-Klinikum, Augustenburger Platz 1, 13353 Berlin, Germany; 10 Austrian Alliance of Lactation Consultants, Ortsstraße 144/8/2, 2331 Vösendorf, Austria; 11 Lithuanian Lactation and Breastfeeding Consultants Association, Pušų str.32, 08116 Vilnius, Lithuania; 12 IBFAN Luxembourg, 17 rue Charlemagne, 1328 Luxembourg, Luxembourg; 13 Breastfeeding Promotion Network of India (BPNI), BP-33 Pitampura, Delhi, 110034 India

**Keywords:** Global strategy, Infant and young child feeding, Breastfeeding, Europe, WBT*i*, Health policy

## Abstract

**Background:**

To protect children’s right to optimal nutrition, WHO/UNICEF developed a Global Strategy for Infant and Young Child Feeding, endorsed by all 53 WHO/EURO Member States. The World Breastfeeding Trends Initiative (WBT*i*) is a tool for monitoring implementation of the Global Strategy. It comprises 15 indicators, ten referring to policies and programmes, and five to feeding practices. Each is scored on a scale of 10, giving a total score of 150 for Global Strategy implementation. To date, 18 WHO/EURO Member States – Armenia, Austria, Belgium, Bosnia and Herzegovina, Croatia, France, Georgia, Germany, Italy, Lithuania, North Macedonia, Malta, Moldova, Portugal, Spain, Turkey, Ukraine and United Kingdom – have conducted a WBT*i* assessment and produced a report.

**Methods:**

Between June 2018 and May 2019, all 18 WBT*i* European reports were carefully read and analysed by a group of national WBT*i* coordinators. Descriptive data analysis, including inter-country comparisons, was conducted using frequencies and percentages. This paper summarises the findings. The full 88-page report will be published on the WBT*i* website.

**Results:**

Three-quarters of 18 European countries have adequate maternity protection, and two-thirds have breastfeeding initiation rates of 50% or higher. However, ‘Preparedness and planning for appropriate and safe Infant and Young Child Feeding (IYCF) in emergencies’ is seriously neglected. Breastfeeding duration is far below WHO recommendations, with an average of 8.7 months. Only three European countries have a budget allocated for implementing IYCF policies and plans, and a third currently have no Baby-friendly designated maternity facilities. Bottle feeding is prevalent, despite its inherent risks, monitoring of IYCF practices is inadequate, with most countries not routinely collecting data, and violations of the International Code of Marketing of Breast-milk Substitutes are commonplace.

**Conclusions:**

European governments are not doing enough to protect, promote and support sound infant and young child feeding practices. Political commitment at the highest level and adequate funding are required to ensure optimal IYCF for Europe’s babies. This report highlights worrying gaps, thereby providing governments, international organisations and other concerned parties with an opportunity to invest in priority areas and, by doing so, hopefully create a better future for our babies.

## Background

In May 2002, the Member States of the World Health Assembly (WHA) – the world’s highest health-policy setting body – renewed their commitment to supporting appropriate infant and young child nutrition, in particular breastfeeding, by unanimously endorsing the Global Strategy for Infant and Young Child Feeding (Global Strategy) [1]. This seminal document, jointly developed by the World Health Organisation (WHO) and the United Nations Children’s Fund (UNICEF), calls upon governments, international organisations and other concerned parties to implement optimal infant and young child feeding (IYCF) policies, programmes and practices, at a national level, using an integrated, comprehensive approach (Table 1).

**Table 1:** Key features of the Global Strategy for Infant and Young Child Feeding

**Aims:**	
• Improve – through optimal feeding – the nutritional status, growth and development, health, and thus the survival of infants and young children.	
• Create an environment that will enable mothers, families and other caregivers, to make – and implement – informed choices about optimal feeding practices.	
**Operational targets:**	
• Appoint a national breastfeeding committee and coordinator.	
• Ensure all maternity facilities implement the Baby-friendly Hospital Initiative (BFHI).	
• Expand the BFHI to include clinics, health centres and paediatric wards.	
• Uphold the International Code of Marketing of Breast-milk Substitutes.	
• Protect and enforce the breastfeeding rights of working women.	
• Regular monitoring of feeding practices.	
• Develop, implement, monitor and evaluate a comprehensive IYCF policy.	
• Protect, promote and support exclusive breastfeeding for 6 months and continued breastfeeding up to 2 years of age or beyond.	
• Promote timely, adequate, safe and appropriate complementary feeding.	
• Provide guidance on IYCF in exceptionally difficult circumstances, e.g. natural catastrophes or in the setting of HIV.	
• Ensure all those communicating with the general public, including educational and media authorities, provide accurate and complete information on IYCF.	
• Ensure skilled counselling is provided to mothers by training health workers and revising pre-service curricula.	
• Enable breastfeeding dyads to stay together during hospitalisation.	
• Develop community-based IYCF support networks, e.g. mother-to-mother support groups.	

Additional impetus to improve maternal and IYCF was provided in 2012 when six global nutrition targets for 2025 were specified and endorsed by the WHA, including “increase the rate of exclusive breastfeeding in the first 6 months up to at least 50%” [2]. In 2017, 40% of babies globally were exclusively breastfed for at least 6 months [3], with the European Region having the lowest prevalence of all six WHO regions – 23% [4]. Given the high prevalence of childhood obesity in Europe and its association with never having been breastfed or being breastfed for less than 6 months [5], in addition to the significant economic losses associated with suboptimal IYCF [6], governments need to re-focus their attention on implementing the Global Strategy, as an evidence-based means of improving health and prosperity, in line with the Sustainable Development Goals for 2030.

Monitoring of policy implementation and programmes and their evaluation at regular intervals is essential for improving both the policy itself and its implementation. Regular monitoring of optimal IYCF practices can help identify improvements and gaps as well as action that needs to be carried out to enhance these practices. The World Breastfeeding Trends Initiative (WBT*i*) was launched globally in 2008 to monitor implementation of policies, programmes and practices recommended in the Global Strategy. At present, 97 countries have conducted a WBT*i* assessment and published a report [7]. The aim of this study is to assess the progress that has been achieved towards implementation of the Global Strategy in Europe using the WBT*i* tool.

## Methods

A detailed description of the methods used, with an in-depth analysis of individual country results including best practice examples, will be published in the full report on the WBT*i* website.

The WBT*i* tool, based on the Global Strategy, consists of 15 indicators used in assessing country IYCF policies, programmes and practices (Table 2). Each indicator is scored on a scale of 10, giving a total score of 150 on Global Strategy implementation – 100 for policies and programmes and 50 for practices. Scores are colour-coded (red, yellow, blue or green) to aid interpretation of results and ranking of scores. To date, 18 countries in the WHO European Region have produced and published a country report, involving at least 154 partners. Each country report is produced in a standardised way according to the WBT*i* process. Firstly, a national coordinator is selected who, following a three-day training course, forms a group of 4–5 core members/assessors representing government, professional and relevant non-governmental organisations, without conflicts of interests. A WBT*i* Guide Book [8], Assessment Tool [9] and Reporting Templates [10] are used to conduct and report assessments, based on available national data. The preliminary report is sent to relevant national partners. After incorporating feedback, the final report is forwarded to the Global WBT*i* Secretariat for validation. The Report, and accompanying summary Report Card, is published on the WBT*i* website, and findings are shared with the wider audience, including government officials and professional organisations, via a ‘Call to Action’. Re-assessment is encouraged every 3–5 years to track trends, assess progress and study the impact of any particular intervention.

**Table 2:** World Breastfeeding Trends Initiative Indicators

Part I: policy and programmes (Indicator 1–10)	Part II: infant feeding practices (Indicator 11–15)
1. National Policy, Programme and Coordination 2. Baby-friendly Hospital Initiative 3. Implementation of the International Code of Marketing of Breastmilk Substitutes 4. Maternity Protection 5. Health and Nutrition Care Systems 6. Mother Support and Community Outreach 7. Information Support 8. Infant Feeding and HIV 9. Infant Feeding During Emergencies 10. Monitoring and Evaluation	11. Early Initiation of Breastfeeding 12. Exclusive Breastfeeding 13. Median Duration of Breastfeeding 14. Bottle Feeding 15. Complementary Feeding

In June 2018, several European WBT*i* coordinators decided to conduct an overview of Global Strategy implementation in the WHO European Region. Between June and October 2018, each coordinator carefully read all 18 published WBT*i* reports and presented the findings for their chosen indicator/s under the following headings: Background, Key Question, Criteria for Assessment, Findings and Detailed Findings. In addition, Key Findings, Key Recommendations and Best Practices were highlighted. The ‘Best Practice’ scenarios, new to the report, represent real-world examples of what European countries have done to improve Global Strategy implementation. Between October 2018 and June 2019, several iterations of the Report were produced, involving contacting national WBT*i* teams for clarification of findings, standardising reporting format and achieving consensus on Report content. This paper represents a summary of these findings.

## Results

### Overall implementation of the global strategy in the WHO European region

**Fig. 1:**
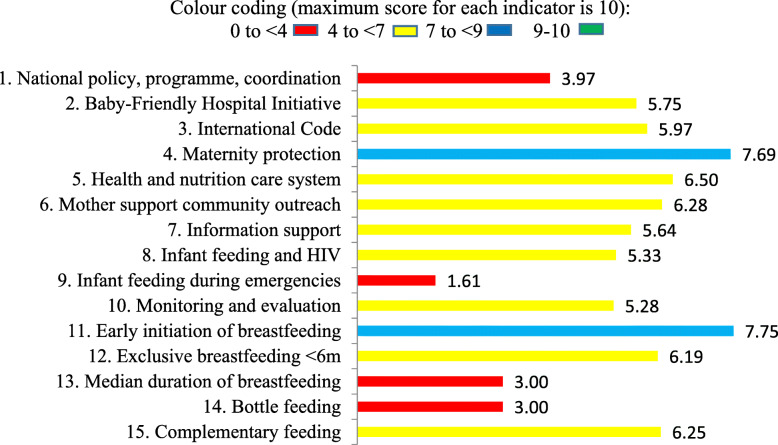
Average colour-coded scores for ten policy/programme and five practice indicators in 18 European countries

The top five ranking countries, in descending order, are Turkey, Croatia, Ukraine, Portugal and Georgia, and the five countries with the lowest level of Global Strategy implementation are Austria, Germany, Lithuania, Belgium and Spain, all within the European Union (Table 3). Figure 1 shows average scores for each of the ten policy/programme and five practice indicators; the overall average score is 5.4. By far the weakest score is ‘Preparedness and planning for appropriate and safe IYCF in emergencies’, with an average of 1.6 out of 10. Table 4 shows individual country scores on policy and programme indicators. Practice indicators fared worse than policy/programme indicators; the lowest score was obtained for median duration of breastfeeding. Despite WHO’s recommendation to breastfeed to age 2 years or beyond, the average duration of breastfeeding in the 13 European countries with available data was 8.7 months. Similarly, ‘Avoidance of bottle feeding’ ranked very poorly, indicating that bottle feeding is a prevalent practice in Europe, despite its inherent risks [11].

**Table 3:**
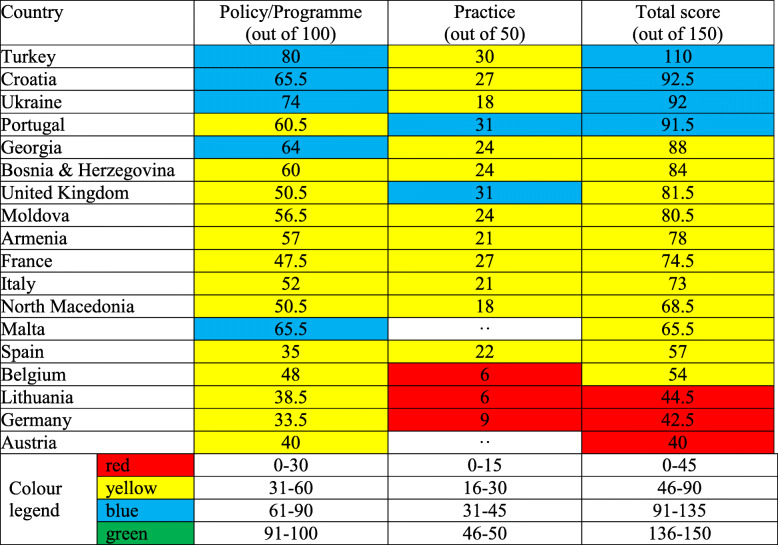
Colour-coded scores for ten policy/programme and five practice indicators in 18 European countries

**Table 4:**
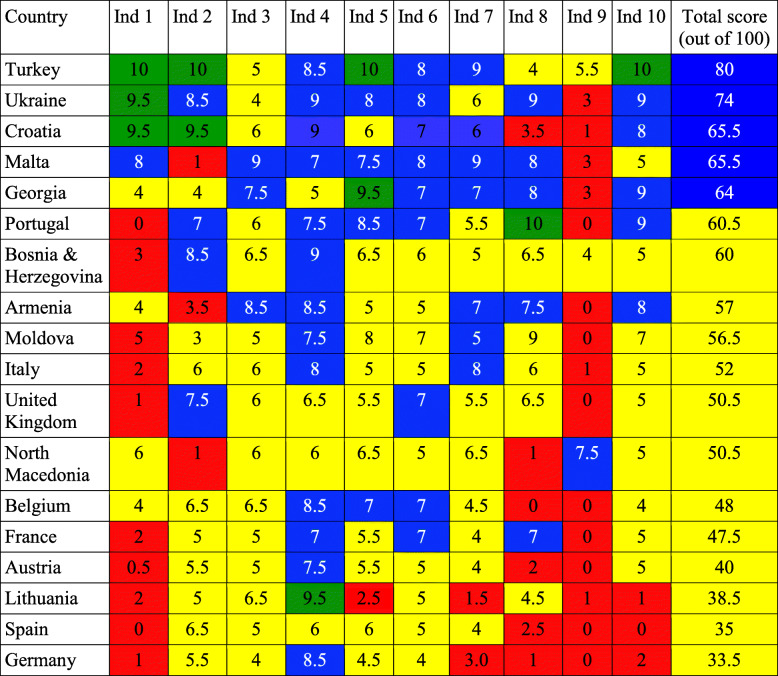
Country scores on policy and programme indicators

### Indicator 1: national policy, programme and coordination

The Global Strategy calls upon governments to adopt a national IYCF policy, which adheres to WHO infant feeding recommendations, based upon which a national action plan is developed and adequately funded. This should be coordinated by a multi-sectoral National Breastfeeding Committee (NBC), led by a coordinator, that meets regularly and links effectively with other relevant sectors. Turkey is the only country that has been assessed as fully implementing this indicator, with Ukraine and Croatia also receiving high scores. Eleven countries (61%) have an official IYCF policy of which all but the United Kingdom adhere to WHO infant feeding recommendations. Eight countries (44%) have a national plan of action, but only three stated it is adequately funded (Turkey, Croatia, Ukraine). All but eight countries (Armenia, Georgia, France, Italy, United Kingdom, Austria, Portugal and Spain) have an NBC, but members only meet regularly in six countries (Turkey, Croatia, Ukraine, Malta, North Macedonia and Georgia). Five of 18 countries (Turkey, Croatia, Ukraine, Malta and Belgium) have a national breastfeeding coordinator with clear terms of reference who regularly communicates national policy to other sectors. Interestingly, all countries scoring less than 4 out of 10 points for this indicator were in the European Union when the reports were written, except for Bosnia and Herzegovina; they include France, Italy, Lithuania, Germany, United Kingdom, Austria, Portugal and Spain.

### Indicator 2: baby-friendly hospital initiative

**Fig. 2:**
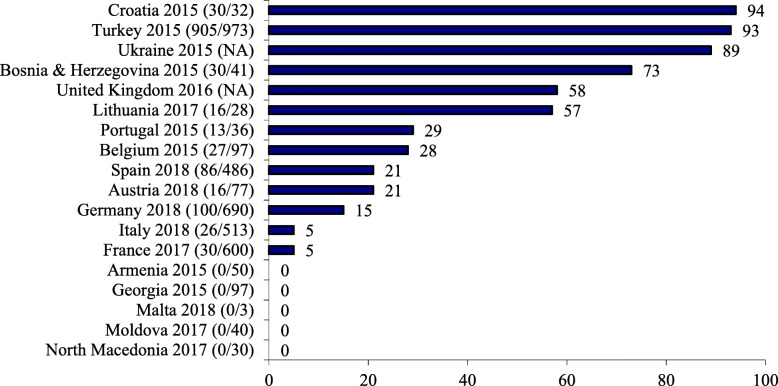
Number (n/N) and percentage (bar) of maternity units recognised as Baby-friendly in each country

The Global Strategy states that every facility providing maternity services should fully implement the BFHI and by doing so ensure that hospital routines and procedures are supportive of timely initiation and establishment of breastfeeding. Evaluation encompasses a quantitative score indicating the percentage of hospitals designated or re-designated in the previous 5 years, and eight qualitative criteria covering training, monitoring, assessment, reassessment, timeliness, HIV integration and use of global criteria. Almost one third of assessed countries (Georgia, Armenia, Moldova, North Macedonia and Malta) do not have any recently designated ‘Baby-friendly’ facilities (in Armenia, 22 facilities were designated between 1999 and 2008, but after 2008 the BFHI was discontinued and reassessments were not conducted), and five countries have over 50% (Turkey, Croatia, Ukraine, UK and Bosnia and Herzegovina), of which Turkey and Croatia have implemented BFHI in over 89% of facilities. Coverage of Baby-friendly facilities in the 13 countries that have introduced BFHI ranges between 5 and 94%. Figure 2 shows the numbers and percentages of maternity units recognised as Baby-friendly in each country. Almost all countries conduct the standard UNICEF/WHO 20-h training course for maternity staff – with the exception of Moldova (although the UK uses a competency-based approach rather than 20 h of training) – but only eight countries (44%) have integrated HIV recommendations into their BFHI programme, representing the criterion with the lowest score. Assessment of BFHI implementation includes interviewing health personnel and mothers in all but two countries (North Macedonia and Malta), with seven of 18 European countries (Austria, Lithuania, Georgia, Armenia, Moldova, North Macedonia and Malta) having no reassessment process in place, endangering the sustainability of the initiative. All countries, except Bosnia and Herzegovina, Lithuania, North Macedonia and Malta, have a time-bound programme, and global BFHI criteria have been adhered to by most countries, with France, Lithuania, Macedonia and Malta being exceptions.

### Indicator 3: implementation of the international code of Marketing of Breastmilk Substitutes

The International Code was adopted by the WHA in 1981 to protect children and families from unethical marketing of breast milk substitutes. It also ensures that families have access to accurate information about breastfeeding and about the safe and appropriate use of breast milk substitutes. Importantly, the International Code applies to governments, manufacturers and distributors of breast milk substitutes, health workers (both professional and volunteer) and the health system and calls on them all to avoid conflicts of interests. Indicator 3 of the WBT*i* assesses the degree to which the International Code is implemented in national law and how effectively the country is monitoring and enforcing those provisions. None of the 18 European countries has fully implemented the International Code and subsequent WHA resolutions. Malta and Armenia received the highest scores, whereas Germany and Ukraine received the lowest. Half of the countries have a monitoring system in place and all but three report having measures which provide for fines to be imposed on violators. Only four countries (22%) report violations to concerned agencies, with Armenia and Turkey having actually fined companies for violations in the last 3 years. Conflicts of interest and promotion of breast milk substitutes, through the health system, is common throughout Europe, endangering the health and wellbeing of mothers and babies.

### Indicator 4: maternity protection

The Global Strategy calls on governments to protect and enforce the breastfeeding rights of working women, both in the formal and informal sectors. This is essential if women are to follow WHO infant feeding recommendations. The WBT*i* tool assesses key elements of maternity protection, encompassed by the International Labour Organisation (ILO) standards [12]. Overall the situation is good in Europe, with none of the 18 countries assessed providing less than 14 weeks of paid maternity leave in the formal sector, but only seven countries (39%) provide at least 26 weeks, necessary for mothers to exclusively breastfeed for 6 months. Encouragingly, all countries – except for the United Kingdom – allow at least one breastfeeding break or a reduction of hours for working breastfeeding mothers. Of those, only France and Malta do not pay mothers during the break. In the private sector, all countries ensure a minimum of 14 weeks paid maternity leave and breastfeeding breaks, except for Malta, France, UK and Spain, which offer only the paid maternity leave. Turkey is the only country that ensures a workplace space for breastfeeding/expressing and childcare, in the formal sector; seven countries (39%) ensure neither, making this the most poorly adhered-to recommendation. In the informal sector, all countries provide at least some protective measures for working mothers, the exceptions being Germany, North Macedonia and Georgia. The important role of fathers is being increasingly recognised; hence, all but two countries (Austria and North Macedonia), provide at least 3 days’ paternity leave, in both public and private sectors. Pregnant and breastfeeding women are protected by legislation from potentially harmful working conditions in all countries except Georgia, and there is legislation prohibiting discrimination against breastfeeding women in all countries except Spain.

### Indicator 5: health and nutrition care systems

For a system, such as a hospital, to provide good care, it is essential that staff are adequately trained to support mothers and their babies in optimal IYCF practices. WBT*i* indicator 5 explores whether care providers in these systems undergo skills training, both pre- and in-service, whether they receive training on the International Code, whether ‘mother-friendly’ care guidelines have been disseminated [13], and whether hospitals enable breastfeeding dyads to remain together when one of them is hospitalised. According to the 18 available country reports, 13 countries (72%) provide inadequate pre-service training of health care providers in IYCF, whereas just over half provide adequate in-service training. Of concern is that only two countries (Turkey and Ukraine) adequately train health workers on their obligations under the International Code, explaining the high prevalence of International Code violations within the health system throughout Europe. Adherence to mother-and-baby-friendly guidelines ensures that every woman and her newborn are protected from unnecessary practices that are not evidence-based, and are not respectful of their culture, bodily integrity, and dignity. Two-thirds of assessed countries have not disseminated mother-friendly guidelines to all facilities and personnel providing maternity care. Ten countries (56%) do not have adequate policies which enable mothers and babies to stay together when one is hospitalised, especially when the mother is hospitalised.

### Indicator 6: mother support and community outreach

The Global Strategy recognizes the need for community-based support for pregnant and breastfeeding women. Women’s feeding decisions are not taken and carried out in isolation but are influenced by family, friends and the wider community. No mother should breastfeed alone: support for breastfeeding is a collective responsibility. Mothers who breastfeed need time, space and resources to support their decision. Thus women require timely, respectful support and accurate information on IYCF to help build confidence and resolve problems, if they are to continue breastfeeding. In only half of the European countries do all pregnant women have access to community-based antenatal and postnatal IYCF support, whereas in six countries (Georgia, Malta, Moldova, Portugal, Turkey, Ukraine) there is adequate support available at birth, which is particularly important for timely initiation of breastfeeding. In only three countries – Croatia, Moldova, Ukraine – are community-based support services, such as Mother Support Groups, integrated into an overall IYCF policy. Training of community-based volunteers and health workers is the most poorly implemented criterion, with only Belgium providing adequate training on IYCF skills to community workers.

### Indicator 7: information support

Accurate and complete information on nutrition, free from commercial interests, is crucial for mothers and families to make informed decisions about feeding their infants and young children. To ensure this, the Global Strategy calls upon countries to develop strategies for Information, Education and Communication (IEC) on IYCF, utilising various forms of media to reach all sectors of society. To be effective, this should – ideally – be coordinated by a National Breastfeeding Committee and/or Breastfeeding Coordinator, in collaboration with national health authorities. Of the 18 assessed European countries, only seven (Turkey, Malta, Italy, Armenia, Ukraine, Croatia and Portugal) were found to have a national strategy that ensures all IYCF materials are free from commercial influence. Similarly, only seven countries (39%) reported including information in IYCF materials/messages on the risks of artificial feeding; they are Turkey, Malta, Georgia, Armenia, North Macedonia and Moldova. Information on the safe preparation of powdered infant formula, in line with WHO/FAO guidelines [14], is provided in six countries (Turkey, Malta, Italy, Georgia, North Macedonia and the United Kingdom), meaning that 12 European countries do not include this vital information in their IYCF materials. Despite a Cochrane systematic review showing that when breastfeeding support is offered to women, the duration and exclusivity of breastfeeding is increased [15], individual counselling on IYCF is fully provided through the national health system in only half of the assessed countries, and group education and counselling services are widely available in only five (Turkey, Georgia, Ukraine, Croatia and Austria). IYCF activities, such as commemorating World Breastfeeding Week, are being implemented at a local level and are free from commercial interests in less than half the countries assessed.

### Indicator 8: infant feeding and HIV

According to WHO, breastfeeding women living with HIV are advised to exclusively breastfeed their infant for the first 6 months, and then continue breastfeeding until at least 12 months of age, regardless of whether they live in developed or developing countries [16]. It reduces the risk of HIV transmission by about half when antiretroviral (ARV) therapy is not available. However, ARVs given during breastfeeding can reduce the transmission of HIV to as low as 1%. WBT*i* indicator 8 explores whether policies and programmes are in place to ensure that HIV-positive mothers are supported to carry out recommended infant feeding practices. Nine European countries (50%) reported including the topic of infant feeding and HIV in their national IYCF policy, of which only five (Portugal, Moldova, Ukraine, Georgia and Armenia) give effect to the International Code. Only a third of the countries provide training to health staff and community workers on HIV and infant feeding policies, the risks associated with various feeding options and how to provide counselling and support. Other countries do so only partially, as part of the BFHI. Voluntary and confidential HIV testing and counselling should be offered routinely to all couples who are considering pregnancy, as well as to pregnant women and their partners. Four countries, all in the European Union, report that this service is not available, with another three only partially. The majority of countries provide at least some degree of counselling and follow-up to HIV-positive mothers and support to ensure adherence to ARVs, the exceptions being Belgium and Germany. Despite WHO recommendations, only two countries – Portugal and Spain – undertake special efforts to counter misinformation on HIV and IYCF, and promote/support 6 months of exclusive breastfeeding in the general population. Very few countries (Portugal, Moldova, Ukraine) have monitoring systems in place to determine the effects of interventions to prevent HIV transmission through breastfeeding on infant feeding practices and overall health outcomes for mothers and infants, including those who are HIV-negative or of unknown status.

### Indicator 9: infant feeding during emergencies

According to the national WBT*i* reports for 18 countries, Europe is turning a blind eye to the likelihood of emergencies occurring in its territory and the inherent risks to mothers and children where optimal IYCF practices are not protected, promoted and supported (Fig. 1). With global climate and trade changes resulting in an increasing frequency of natural disasters and pandemics, it is essential for countries to be prepared for this challenge and a key component is the inclusion of guidelines on safe IYCF. The Operational Guidance on Infant Feeding in Emergencies provides such guidance [17]. Despite this, only one country – North Macedonia – has a national policy on IYCF in emergencies (IYCFE) that contains all the basic components of the Operational Guidance. Similarly, North Macedonia is the only country that has appointed a person tasked with responsibility for national coordination of IYCFE with relevant partners. Every country is expected to have an emergency-preparedness and response plan including interventions that create an enabling environment for breastfeeding, such as counselling by appropriately trained counsellors, support for re-lactation and wet-nursing, and protected spaces for breastfeeding; again, only North Macedonia fully complies with this recommendation. Measures to minimize the risks of artificial feeding, including an endorsed statement on avoidance of donations of breast milk substitutes, bottles and teats, have been undertaken by only two countries (11%). Lack of allocated resources weakens the ability of a government to act in emergency situations; Turkey was the one country to report adequate resources for implementation of their emergency preparedness and response plan. Relevant health care personnel need to be trained for emergency management; not a single country reported IYCFE being fully integrated into pre- and in-service training of relevant health care personnel and emergency management staff, making this the most poorly rated criterion. North Macedonia was the only country to report that adequate orientation and training is taking place as per the national plan.

### Indicator 10: monitoring and evaluation

Monitoring of policy and programme implementation and evaluation at regular intervals is essential to improve both the policy itself and its implementation. Equally, regular monitoring of IYCF practices can help identify improvements and gaps, as well as action needed to enhance these practices. Indicator 10 of the WBT*i* assesses whether monitoring and evaluation systems are in place, at a national level, to collect, analyse and use routine data in order to improve IYCF practices. Four countries (Turkey, Georgia, Portugal, Ukraine) have built monitoring and evaluation components into major IYCF programme activities, whereas five countries (28%) have integrated monitoring of IYCF practices into their national nutritional surveillance system and/or health information system. Data on progress made in implementing IYCF programme activities are routinely collected at the sub-national and national levels in five countries (Turkey, Georgia, Portugal, Armenia, Croatia). These data are reported to key decision-makers in seven countries (39%) but are used by programme managers to guide planning and investment decisions in only five (Turkey, Portugal, Ukraine, Armenia, Croatia).

### Indicator 11: early initiation of breastfeeding

WBT*i* indicator 11 endeavours to determine, based on nationally available data, the proportion of children born in the last 24 months who were put to the breast within one hour of birth. Data were available for 12 countries, given that a third of the assessed countries do not record the time of initiation of breastfeeding. Wide regional variability exists in reported initiation rates, ranging between 21% in North Macedonia to 84% in Portugal, with an average rate of 57%, which is comparable to the reported rate for Eastern Europe and Central Asia of 56% [18].

### Indicator 12: exclusive breastfeeding for the first 6 months

WBT*i* indicator 12 calculates the percentage of babies 0–5.9 months of age who are exclusively breastfed, based on 24-h recall. Rates vary widely across the WHO European Region Member States, with the lowest rate in France (10%) and the highest in Croatia (65%). Interestingly, Croatia has one of the highest proportions of ‘Baby-friendly’ maternity facilities in the European region and offers 12 months of paid maternity leave. Overall, the rate of exclusive breastfeeding for infants under 6 months of age in the 16 countries which provided data for this indicator was 40%.

### Indicator 13: median duration of breastfeeding

Indicator 13 was the most poorly rated IYCF practice in the 14 Member States of the WHO European Region which provided data. Median breastfeeding duration rates varied drastically, between 3 months (United Kingdom) and 17 months (Turkey), with higher rates found in less developed and non-EU countries. The average median duration of breastfeeding in the assessed countries was 8.7 months, far below the recommended 24 months. In only three countries – Georgia, Moldova and North Macedonia – does it even reach the age of 1 year, with only Turkey exceeding this. Interestingly, in Turkey, the median duration of breastfeeding among girls and boys differs, with boys being breastfed approximately 2 months longer than girls.

### Indicator 14: bottle feeding

WBT*i* indicator 14 endeavours to determine the proportion of babies 1–12 months of age who are fed with any foods or drinks (including breast milk) from a bottle. Data were available for only nine countries, of which five used indirect data to provide an estimate. The average rate of bottle feeding for the remaining four countries (Armenia, Moldova, Portugal, Turkey) was found to be 58%, indicating that bottle feeding is a prevalent practice in Europe.

### Indicator 15: complementary feeding

WBT*i* indicator 15 endeavours to determine the proportion of breastfed babies receiving complementary foods between 6 and 9 months of age. Wide inter-country variability was noted in the WHO European Region, with North Macedonia rating this indicator at 28%, and Portugal, at the other end, recording 100%. Large variation was also noted within countries, with some mothers commencing complementary foods as early as 7 days and others as late as 305 days (France). The median age of introduction of solid foods was found to be related to breastfeeding duration, with children who were never breastfed starting solids earlier (136 days) than children breastfed at least 4 months (166.5 days).

## Discussion

Despite unanimous support from the Member States of the World Health Assembly for implementation of the Global Strategy for Infant and Young Child Feeding, our analysis of individual country reports from 18 European countries indicates that European governments are not doing enough to fulfil this commitment. Preparedness and planning for appropriate and safe IYCF in emergencies is seriously neglected; breastfeeding duration is far below WHO recommendations; a third of assessed countries have no Baby-friendly designated maternity facilities; bottle feeding is a prevalent practice; monitoring of IYCF practices is at best patchy, and violations of the International Code of Marketing of Breast-milk Substitutes are commonplace.

A closer look at individual country scores on IYCF policy and programme indicators reveals that the countries scoring most highly all score well on Indicators 1 (National policy, programme and coordination) and 10 (Monitoring and evaluation). Only the three countries with the highest total scores - Turkey, Ukraine and Croatia - reported that the national plan of action is adequately funded. The five countries that reported programme managers use national data to guide planning and investment decisions are all among the eight countries with the highest total scores. These findings highlight the importance of having national IYCF programmes that are well-coordinated, monitored and adequately resourced, both financially and with enough well-trained staff. Interestingly, the five countries with the lowest level of Global Strategy implementation are Austria, Germany, Lithuania, Belgium and Spain, all within the European Union, suggesting European Union governments may not be investing adequate resources to produce measurable results. Considering that every dollar invested in breastfeeding generates 35 US dollars in economic returns [19], the European Union urgently needs to reconsider its IYCF policies.

The Baby-friendly Hospital Initiative has been shown to positively impact breastfeeding outcomes, with a dose–response relationship found between the number of ‘Steps’ a mother is exposed to and the likelihood of improved breastfeeding practices [20]. Despite this, low levels of implementation have been observed worldwide with only 10% of infants in 2017 being born in a ‘Baby Friendly’ facility. Obstacles to BFHI implementation include aggressive marketing of breast milk substitutes, lack of recognition and support of BFHI by governments, and inadequate monitoring and reassessment of Baby-friendly facilities [21]. This is illustrated in our study of Armenia, where 22 facilities were designated between 1999 and 2008, but no reassessments were conducted thereafter. This waste of resources must be prevented [22]. Governments need to find a way of sustaining the BFHI. This requires a national BFHI programme, with dedicated national leadership, integrated more fully into the healthcare system. In addition to Armenia, we found four other countries in the European region with no Baby-friendly maternity facilities, whereas in those countries where BFHI is active, coverage of maternity facilities can be as low as 5 %, leaving families in Europe with little choice when it comes to maternity care. Hopefully, the recent revision of Baby-friendly guidance will reinvigorate the BFHI programme, enabling maternity facilities to successfully implement critical management procedures and key clinical practices to make their facilities truly “Baby-friendly” [23].

It is clear that the poor results in Europe for the practice indicators derive from poor policies and programmes and from poor implementation. The WBT*i* has been shown to improve policies and programmes [24]. Full implementation of the International Code and subsequent relevant WHA resolutions is a basic protective measure for breastfeeding and timely complementary feeding. Current legislation, in most European countries, covers only infant formula for use to 6 months, but allows widespread marketing for follow-on and toddler formula, and no restrictions at all on the marketing of feeding bottles and teats. This situation allows manufacturers to circumvent the International Code, for example by using cross-branding and line extension strategies [25], and to keep sponsoring educational events for health professionals, despite the good example set by the Royal College of Paediatrics and Child Health [26].

Our findings may have been affected by problems associated with data collection. Bottle feeding is rarely monitored in Europe; data were available for only nine countries, five using indirect data. Yet, our conclusion that bottle feeding is a prevalent practice is supported by a report showing that Europe is the second largest market worldwide [27]. Some Indicators use objective criteria, as in Indicators 1–4 and 10, whereas some rely, at least partly, on more subjective criteria. For example, standards and curricula in Indicator 5 are only a guide to what is actually covered in a particular course and what is examined. However, by considering a number of training courses for different professions, a general picture emerges. In Indicator 6, access to counselling support is mentioned in two criteria; the quality of the support and extent to which mothers actually access it would be very difficult to assess, but again these criteria contribute to a general picture. Also, each country carries out its own WBT*i* assessments so there may be variability between assessors. This is minimised by providing standard templates, assessment tools and a guide book, and ensuring national WBT*i* coordinators complete a three-day training course before commencing assessment.

Differing interpretations of the WBT*i* indicator wording by country assessors are an additional challenge and may be partly responsible for differing results; a clear example is provided in relation to timely complementary feeding (Indicator 15). Countries such as Portugal interpreted it as the percentage of infants *consuming* solids between 6 and 9 months, whereas other countries, e.g. Croatia, interpreted this indicator as the proportion of infants *initiating* solids during this period. Given the greater tendency towards overnutrition, rather than undernutrition, among children in Europe, the monitoring of the introduction of solids may be more relevant for this region. In addition, although national coordinators are instructed to use nationally representative data for assessment of indicators, this is not always available, and hence presented regional or local results may not be reflective of the true situation in a country. Finally, the lack of available data from Scandinavian countries, where optimal infant and young child feeding is commonly practised, may have created a “differential selection bias”. We hope that the remaining 35 countries from the WHO European region that have not conducted a WBT*i* assessment will join the WBT*i* and, by doing so, contribute to a global repository of data on IYCF which will enable countries to learn from each other and strategically invest resources to remove the barriers to optimal IYCF. For this ideal situation to occur, funding is needed to train and support national WBT*i* coordinators.

## Conclusion

The key problem, underlying all others, is the lack of proper policies, programmes and coordination (Indicator 1). Only three of 18 European countries have a budget allocated for implementing IYCF policies and plans and less than a third has a National Breastfeeding Committee, led by a Coordinator, that meets regularly and collaborates with other relevant sectors. Therefore, there is an urgent need for governments to develop or update a comprehensive, cross-sectoral, multi-level IYCF policy and plan and ensure an adequate budget for its implementation. In addition, governments need to appoint a conflict of interest-free national committee and coordinator, with clear terms of reference, to oversee the implementation of the plan if the Global Strategy is to be successfully implemented and children’s rights to the best possible start in life are to be respected [28]. As stated in the Global Strategy, “Success … rests first and foremost on achieving political commitment at the highest level and assembling the indispensable human and financial resources” [1].

## Data Availability

The datasets analysed during the current study are available on the WBT*i* website: https://www.worldbreastfeedingtrends.org/

## References

[1] WHO (2002). Global strategy for infant and young child feeding.

[2] Global nutrition monitoring framework: operational guidance for tracking progress in meeting targets for 2025. World Health Organization, Geneva, 2017.

[3] Global Breastfeeding Collective (2017). Global breastfeeding scorecard 2017: tracking progress for breastfeeding policies and programmes.

[4] Bosi AT, Eriksen KG, Sobko T, Wijnhoven TM, Breda J (2016). Breastfeeding practices and policies in WHO European region member states. Public Health Nutr.

[5] Rito AI, Buoncristiano M, Spinelli A, Salanave B, Kunesova M, Hejgaard T (2019). Association between characteristics at birth, breastfeeding and obesity in 22 countries: the WHO European childhood obesity surveillance initiative – COSI 2015/2017. Obes Facts.

[6] Rollins NC, Bhandari N, Hajeebhoy N, Horton S, Lutter CK, Martines JC (2016). Why invest, and what it will take to improve breastfeeding practices?. Lancet.

[7] World Breastfeeding Trends Initiative country reports Available at: https://www.worldbreastfeedingtrends.org/wbti-country-report.php. Accessed 20 Jan 2020.

[8] WBTi (2019) The guide book Available at: https://www.worldbreastfeedingtrends.org/resources/the-guide-book. Accessed 20 Jan 2020.

[9] WBTi (2019). Assessment Tool.

[10] WBTi (2020). Reporting templates.

[11] Stuebe A (2009). The risks of not breastfeeding for mothers and infants. Rev Obstet Gynecol.

[12] ILO (2000). Maternity Protection Convention N. 183.

[13] WHO (2003). Infant and Young Child Feeding: a tool for assessing national practices, policies and programmes.

[14] WHO (2007). Safe preparation, storage and handling of powdered infant formula: guidelines.

[15] McFadden A, Gavine A, Renfrew MJ, Wade A, Buchanan P, Taylor JL et al. Support for healthy breastfeeding mothers with healthy term babies. Cochrane Database Syst Rev 2017, Issue 2. Art. No.: CD001141. 10.1002/14651858.CD001141.pub5 PMC6464485 28244064

[16] WHO, UNICEF (2016). Guideline: updates on HIV and infant feeding: the duration of breastfeeding, and support from health services to improve feeding practices among mothers living with HIV.

[17] IFE Core Group. Infant and young child feeding in emergencies: operational guidance for emergency relief staff and programme managers. Oxford: Emergency Nutrition Network; 2017.

[18] UNICEF, WHO (2018). Capture the moment. Early initiation of breastfeeding: The best start for every newborn.

[19] Global Breastfeeding Collective (2017). Nurturing the health and wealth of nations: the investment case for breastfeeding.

[20] Pérez-Escamilla R, Martinez JL, Segura-Pérez S (2016). Impact of the baby-friendly hospital initiative on breastfeeding and child health outcomes: a systematic review. Matern Child Nutr.

[21] Grgurić J, Zakarija-Grković I, Pavičić Bošnjak A, Stanojević M (2016). A multifaceted approach to revitalising the baby-friendly hospital initiative in Croatia. J Hum Lact.

[22] Harutyunyan SA, Poghosyan KP, Saribekyan KS, Ghazaryan AE (2011). Implementation of baby friendly practices in health care system in Armenia. New Armen Med J.

[23] UNICEF, WHO (2018). Implementation guidance: protecting, promoting and supporting breastfeeding in facilities providing maternity and newborn services – the revised baby-friendly hospital initiative.

[24] Gupta A, Suri S, Dadhich JP, Trejos M, Nalubanga B (2019). The world breastfeeding trends initiative: implementation of the global strategy for infant and young child feeding in 84 countries. J Public Health Policy.

[25] Cattaneo A, Pani P, Carletti C, Guidetti M, Mutti V, Guidetti C (2015). Advertisements of follow-on formula and their perception by pregnant women and mothers in Italy. Arch Dis Child.

[26] Mayor S (2019). Royal college stops taking funding from formula milk firms. BMJ.

[27] The Express Wire (2019). Baby Bottles Market.

[28] United Nations CRC (2013). Committee’s general comments no 15 on the right of the child to the enjoyment of the highest attainable standard of health.

